# Prevalence and Molecular Characterization of Metallo β-Lactamase Producing Gram-Negative Pathogens Causing Eye Infections

**DOI:** 10.3389/fpubh.2022.870354

**Published:** 2022-06-14

**Authors:** Gunasekaran Rameshkumar, Ranjithkumar Dhandapani, Prajna Lalitha, Siva Ganesa Karthikeyan Rajapandian, Velmurugan Palanivel, Sathiamoorthi Thangavelu, Abdullah A. Alyousef, Thamer Albalawi, Pravej Alam, Mohammad Zubair, Fayez M. Saleh, Fuad Abdullah Alatawi, Fohad M. Husain

**Affiliations:** ^1^Medical Microbiology Laboratory, Department of Microbiology, Alagappa University, Karaikudi, India; ^2^Department of Ocular Microbiology, Aravind Eye Hospital, Madurai, India; ^3^Centre for Material Engineering and Regenerative Medicine Bharath Institute of Higher Education, Chennai, India; ^4^Clinical Laboratory Sciences Department, College of Applied Medical Sciences, King Saud University, Riyadh, Saudi Arabia; ^5^Department of Biology, College of Sciences and Humanities, Prince Sattam Bin Andulaziz University, Alkharj, Saudi Arabia; ^6^Department of Medical Microbiology, Faculty of Medicine, University of Tabuk, Tabuk, Saudi Arabia; ^7^Department of Biology, Faculty of Science, University of Tabuk, Tabuk, Saudi Arabia; ^8^Department of Food Science and Nutrition, King Saud University, Riyadh, Saudi Arabia

**Keywords:** Metallo β-lactamases, *Enterobacteriaceae*, non-*Enterobacteriaceae*, normal flora, gram-negative bacilli

## Abstract

**Purpose:**

Metallo β-lactamases (MβL) production is a worldwide problem, particularly in gram-negative bacteria. As scanty data is available on the prevalence of MBL, the present study is being undertaken to determine the prevalence, antibacterial sensitivity patterns, and molecular characterization of MβL associated resistant genes in gram-negative bacteria isolated from ocular infections.

**Material and Methods:**

At a tertiary eye care center in south India, 359 gram-negative pathogens, 200 isolates from eye infections, and 159 isolates from normal flora of the eye were studied. A gold standard microbiology method was used to identify the isolates. An antibiotic double disc synergy test and a combination disc test were used to detect MβL production. Multiplex PCR was used to investigate the molecular characteristics of the MβL encoding genes *bla*_VIM_, *bla*_IMP_, and *bla*_NDM_.

**Results:**

Of the 359 gram-negative bacterial pathogens, *Pseudomonas aeruginosa* 108 (30.1%) and *Enterobacter agglomerans* 46 (12.8%) were commonly isolated. High prevalence of *P. aeruginosa* 81% (17 strains) was detected as an MβL producer and it shows 100% resistance to 2nd and 3rd generation cephalosporins and meropenem. Multiplex PCR detected only the *bla*_VIM_ gene in 56 (28%) of various eye infections and 27 (17%) of normal flora of the gram-negative bacteria (GNB). The *bla*_VIM_ gene is detected predominantly in 51.8% of keratitis and 21.4% of postoperative endophthalmitis. High prevalence of the gene was detected in *P. aeruginosa* 42.9% (24 of 56) and *Alcaligens denitrificans* 10.7% (6 of 56) from eye infections. Whereas, in the control group, *P. aeruginosa* and *E. coli* each had 14.8% (4 of 27) that were shown positive.

**Conclusion:**

The emerging MβLs mediated resistance among *P. aeruginosa* is a challenging task for ophthalmologists, especially in patients with endophthalmitis and bacterial keratitis. This local knowledge will aid in advising appropriate antibiotic use and avoiding unnecessary antibiotic prescriptions, which are highly warranted.

## Introduction

The eye is a unique organ with physical barriers that keep out infectious agents, yet due to the eye's surface invariability, it is susceptible to a vast variety of microbial infections (1). Microorganisms can permeate and damage the internal parts of the eye, which often results in irreversible loss of vison (2). Bacterial infections of the eye are most commonly caused by external sources such as penetrating trauma or by microorganisms conquering the intraocular via the bloodstream (3). Changes in normal flora can also cause external and internal eye infections (1, 4, 5). *Pseudomonas aeruginosa, Enterobacter species, Klebsiella pneumoniae*, and *Alcaligens denitrificans* are the most common gram-negative bacteria that cause eye infections (6). Due to their stability against the majority of β-lactamases and high permeation rate through bacterial outer membranes, the β-lactam groups of antibiotics, such as carbapenem drugs, are potential agents in treating multidrug resistant gram-negative bacterial infections (7). However, the efficacy of these drugs is increasingly being limited because of the emergence of carbapenem-resistant pathogens world-wide, especially in Southeast Asia and Europe (8, 9).

Understanding antibacterial resistance is crucial for selecting appropriate antibiotics to control infection, thus preventing vision-threatening complications. Overuse of antibiotics for systemic infections and topical antibiotics for eye infections are both factors that contribute to the development of antibiotic resistance among ocular isolates (5). Routine antibacterial susceptibility disc diffusion testing, as well as molecular approaches for drug resistance detection, are highly recommended for understanding the incidence of antibiotic resistance to extended-spectrum antibiotics (6). The spread of resistance to β-lactam group antibiotics occurs through β-lactamase enzyme production by gram-negative bacteria and is an emerging problem. β-lactamase are divided into four groups, with categories A, C, and D having serine at their active site, and Metallo- lactamase (MβL) belonging to the Amber class B (10, 11). Most other β-lactam antibiotics, with the exemption of monobactam can be hydrolysed by the MβL generating bacterium, including penicillin, cephalosporin, and carbapenem (10, 11). Bacterial isolates that produce MβLs are also associated with increased morbidity and mortality (12). Mobile genetic components such as transposons, plasmids, and integrons, which have the ability to move both within and between species, contain MβL gene (10, 11).

The prevalence of different types of acquired MβLs genes today can be ascertained rapidly (13). The different forms of MβL genes have been discovered in gram-negative bacteria including (i) Verona integron- coded MβL (VIM), (ii) imipenemase (IMP), Seoul imipenemase (SIM), (iii) Germany imipenemase (GIM), (iv) Spaulo MβL (SPM), (v) New delhi MβL (NDM). VIM and IMP are the most common inherited MβL genotypes in India (14, 15). The incidence of MβLs positive isolates in hospital settings is not only a therapeutic challenge but also a major problem in infection control management. Early detection of the prevalence and types of MβLs is very important, with benefits including timely implementation of strict infection control measures and treatment with different antimicrobials. MβL genes have spread from *Pseudomonas aeruginosa* to the members of the *Enterobacteriaceae* family in recent years (16). In India, the MβLs have been found in a variety of systemic infections, but only one center has reported on ocular infections (17). The purpose of this study was to determine the prevalence and antibacterial susceptibility profile of MβL producing gram-negative bacteria isolated from various eye infections and normal flora of the eye (preoperative conjunctival flora, i.e., control group).

## Materials and Methods

### Bacterial Isolates and Research Subjects

This study only includes gram-negative bacteria isolated from blepharitis, conjunctivitis, contact lens associated keratitis, bacterial keratitis, dacryocystitis, canaliculitis, and endophthalmitis, as well as samples from patients with penetrating and therapeutic keratoplasty surgeries, post suture infection, and normal flora of the eye (preoperative conjunctival swabs collected from patients who underwent cataract surgeries, i.e., control group) between January 2014 and December 2016, who were treated at a tertiary eye care referral center in South India. The study was approved by the institutional review board and follows the tenets of the Declaration of Helsinki. After detailed slit-lamp biomicroscopic examination, specimens were collected by ophthalmologists for direct microscopic observation and cultures. The samples were inoculated onto the 5% sheep blood agar (M450 -Tryptose blood Agar base), chocolate agar, potato dextrose agar (GM096), thioglycollate broth (M009), and brain heart infusion broth (BHI) (M210) separately (Hi-Media Laboratories Pvt. Ltd, Mumbai, India). After inoculation of specimen onto the culture media were incubated at 37°C for the growth of bacteria, chocolate agar were incubated in 3–5% CO_2_ incubator (Thermo Scientific, Model: 3111, HEPA Class 100,USA) at 37°C, Potato dextrose agar were incubated at BOD incubator (Techlab, SZ135, India) at 25–27°C for the growth of fungi. After the growth in the media, the pure culture of the organism was identified up to species level using standard biochemical tests. For this study, only gram-negative bacteria were included for further analysis.

### Antibacterial Susceptibility Testing

The Kirby-Bauer disc diffusion method was used to test antibacterial susceptibility *in vitro* for each pure bacterial isolate, and the results were interpreted using the Clinical and Laboratory Standards Institute's serum standards (CLSI) (2012) (18). The antibacterial agents (BIO-RAD Laboratories, France) used were amikacin (30 μg), tobramycin (10 μg), gentamicin (10 μg), cefazolin (30 μg), cefotaxime (30 μg), ceftazidime (30 μg), cefpodoxime (10 μg), ciprofloxacin (5 μg), ofloxacin (5 μg), levofloxacin (5 μg), gatifloxacin (5 μg), moxifloxacin (5 μg), chloramphenicol (30 μg), imipenem (10 μg), meropenem (10 μg), aztreonam (10 μg), cefepime (30 μg), cefoxitin (30 μg), piperacillin (10 μg), piperacillin/tazobactam (100/10 μg), ticarcillin (75 μg) ticarcillin/clavulanic acid (75/10 μg), ceftriaxone (30 μg), and cefotetan (30 μg). As a reference and quality control, standard American Type Culture Collection (ATCC) bacterial isolates were used (*Pseudomonas aeruginosa* ATCC 27853, *Escherichia coli* ATCC 25922).

### Phenotypic Detection of MβL Activity

#### Combined Disc Test

The combined disc test was carried out as per the method advocated by Yong et al. (19). The isolate was adjusted to the 0.5 McFarland standard by Muller-Hinton (MH) broth and swabbed on the MH agar plates. Imipenem (10 microgram) and meropenem discs (10 microgram) were placed on the inoculated agar plate, and 10 μL of 0.5 M EDTA solution was impregnated onto each antibiotic disc. The plates were incubated overnight at 37°C. After incubation, the zone of inhibition was measured and compared. Imipenem and imipenem integrated with EDTA disc or meropenem and meropenem integrated with EDTA discs showed a zone of inhibition >7 mm which was considered to be positive MβLs production of the isolate.

#### Double Disc Synergy Assay

This assay was conducted based on Arakawa et al. (20) and Lee et al. (21) with modifications. The MH agar plate was inoculated with the isolate of 0.5 McFarland standard. A 10 microgram imipenem disc or a 10 microgram meropenem disc was put down on the agar plate. A sterile disc (6 mm) was impregnated with 10 μL of 0.5 M EDTA solution. These plates were incubated overnight at 37°C. After incubation, the plates were observed for synergistic activity.

### Molecular Characterization of MβL Genes

#### Preparation of Template DNA

A single colony was inoculated into 5 mL of Luria-Bertani broth (Himedia, Mumbai) and incubated for 20 h at 37°C. Cells were harvested from a 1.5 mL culture by centrifugation at 12,000 rpm for 5 min. According to the manufacturer's instructions, DNA was extracted using the QIAamp® DNA micro kit (51304). (QIAGEN, Hilden, Germany). DNA was extracted from the test isolates along with the *bla*_VIM_ gene carrying *P. aeruginosa 1138* (GenBank accession number KC505236) (positive control for *bla*_VIM−2_ gene), *bla*_IMP_ gene carrying *P. aeruginosa* R-61 (GenBank accession number KF570107) (positive control for *bla*_IMP−1_ gene) and *bla*_NDM_ gene carrying *Klebsiella pneumoniae* BC5500 (Genbank accession number KF570106) (positive control for *bla*_NDM−1_ gene). As a negative control, a non-MβL producing *P. aeruginosa* ATCC 27853 was used.

#### Gene-Specific Oligonucleotide Primer Pairs

The gene specific primers, *bla*_VIM_–F- 5′-GATGGTGTTTGGTCGCATA-3′ and *bla*_VIM_-R- 5′ CGAATGCGCAGCACCAG-3′ which amplified a 390 bp amplicon, *bla*_IMP−_ F-5′- TTGACACTCCATTTACDG-3′ and *bla*_IMP_-R-5′-GATYGAGAATTAAGCCACYCT-3′ which amplified a 139bp amplicon as described by Dallenne et al. (22) and *bla*_NDM_ -F-5′-CCAGCTTGCCCCGCAAGAGG-3′ and *bla*_NDM_-R-5′ATCGGGGGCGGAATGGCTCA- 3′ which amplified a 350 bp amplicon were selected for study (22).

#### Multiplex Polymerase Chain Reaction for Bla_VIM_, Bla_IMP_, and Bla_NDM-_Gene Identification

Multiplex PCRs were performed in the Eppendorf Mastercycler ProS (Hamburg, Germany) with a final volume of 25 μL in 0.2 mL thin-walled PCR tubes. Each reaction contained 2.5 μL of 10X PCR buffer (10 mM Tris-HCl, pH 8.3, 1.5 mM MgCl_2_), 0.5 μL dNTP's (10 μM), 1 μL (each) primers (10 pmol/mL) with 0.2 μL (3 U/μl) *Taq* DNA polymerase (Bangalore Genei, Bangalore, India) was added to the reaction mixture. The multiplex PCR cycling conditions for amplification were as follows: The initial denaturation was at 94°C for 5 min and 30 cycles, 40 s at 94°C, 40 s at 55°C, and 1 min at 72°C, followed by a final extension of 7 min at 72°C for *bla*_VIM_, *bla*_IMP_, and *bla*_NDM−_genes. After the amplification reaction, PCR products were analyzed by electrophoresis on 1.5% agarose gels.

## Results

### Bacterial Isolates and Study Subjects

A total of 359 gram-negative bacterial isolates were isolated from 355 patients during the study period. Of which 159 (44%) isolates were from the control group (preoperative conjunctival flora from patients with no infection who underwent cataract surgery), and the remaining 200 (56%) isolates were from various ocular infections. The major disease samples received during the study duration are corneal scrapings from keratitis patients (52 of 200; 26%), followed by contact-lens associated keratitis (36 of 200; 18%), conjunctivitis (29 of 200; 14.5%), and endophthalmitis (26 of 200; 13%) (Table 1). The highest number of gram-negative bacterial species isolated from the control group was *K. pneumoniae* (23 of 159; 14.5%), followed by *P. aeruginosa* (22 of 159; 13.8%), and *E. agglomerans* (21 of 159; 13.2%). In the infection group, *P. aeruginosa* (86 of 200; 43%) was the predominant isolate, followed by *E. agglomerans* (25 of 200; 12.5%), *K. pneumoniae* (16 of 200; 8%) and *E. coli* (11 of 200; 5.5%) (Table 2).

**Table 1:** List of specimens study included based on the diagnosis (*n* = 355).

**Diagnosis**	**Specimen**	**Total (*n =* 355)**
Keratitis (*n =* 121)	Corneal scraping	**52**
	Contact lens solution	**36**
	Corneal button	**30**
	Bandage contact lens	**3**
Conjunctivitis	Conjunctival swab	**29**
Endophthalmitis (*n =* 26)*	Vitreous fluid	**25**
	IOL	**1**
Dacryocystitis	Pus	**12**
Suture infection (*n =* 5)	Suture material	**2**
	Foreign body	**3**
Canaliculitis	Lid abscess	**2**
Blepharitis	Lid swab	**1**
Pre-operative conjunctival	Conjunctival swab	**159**
flora (Control group)

* Post-operative (n = 21), Trauma (n = 2), Post IVTA (n = 1) and Due to keratitis (n = 1). Bold values show the total in numbers for respective category.

**Table 2:** Distribution of gram-negative isolates from various ocular infections and pre-operative conjunctival flora (Control group).

**Name of the bacterial isolates**	**Ocular infections**		**Pre-operative conjunctival flora (Control group)**		**Total**	
	***n***	**%**	***n***	**%**	***n***	**%**
***Non-Enterobacteriaceae***	**127**	**63.5**	**59**	**37.1**	**186**	**51.8**
*P. aeruginosa*	86	43	22	13.8	108	30.1
*P. alcaligenes*	16	8	3	1.9	19	5.3
*A. denitrificans*	10	5	6	3.8	16	4.5
*A. faecalis*	7	3.5	5	3.1	12	3.3
*A. lwoffii*	5	2.5	7	4.4	12	3.3
*A. hydrophila*	3	1.5	16	10.1	19	5.3
***Enterobacteriaceae***	**73**	**36.5**	**100**	**62.9**	**173**	**48.2**
*E. agglomerans*	25	12.5	21	13.2	46	12.8
*K. pneumoniae*	16	8	23	14.5	39	10.9
*E. coli*	11	5.5	13	8.2	24	6.7
*S. marcescens*	10	5	1	0.6	11	3.1
*E. aerogenes*	5	2.5	3	1.9	8	2.2
*C. freundii*	2	1	1	0.6	3	0.8
*K. oxytoca*	2	1	1	0.6	3	0.8
*M. morgonii*	1	0.5	6	3.8	7	1.9
*P. rettgeri*	1	0.5	1	0.6	2	0.6
*P. mirabilis*	0	0	11	6.9	11	3.1
*C. diversus*	0	0	10	6.3	10	2.8
*C. koseri*	0	0	6	3.8	6	1.7
*E. cloacae*	0	0	1	0.6	1	0.3
*P. vulgaris*	0	0	1	0.6	1	0.3
*V. furnissii*	0	0	1	0.6	1	0.3
***Total in numbers (%)***	**200**	**100**	**159**	**100**	**359**	**100**

*Bold values show the total in numbers for respective category*.

### Antibacterial Susceptibility Test

**Figure 1:**
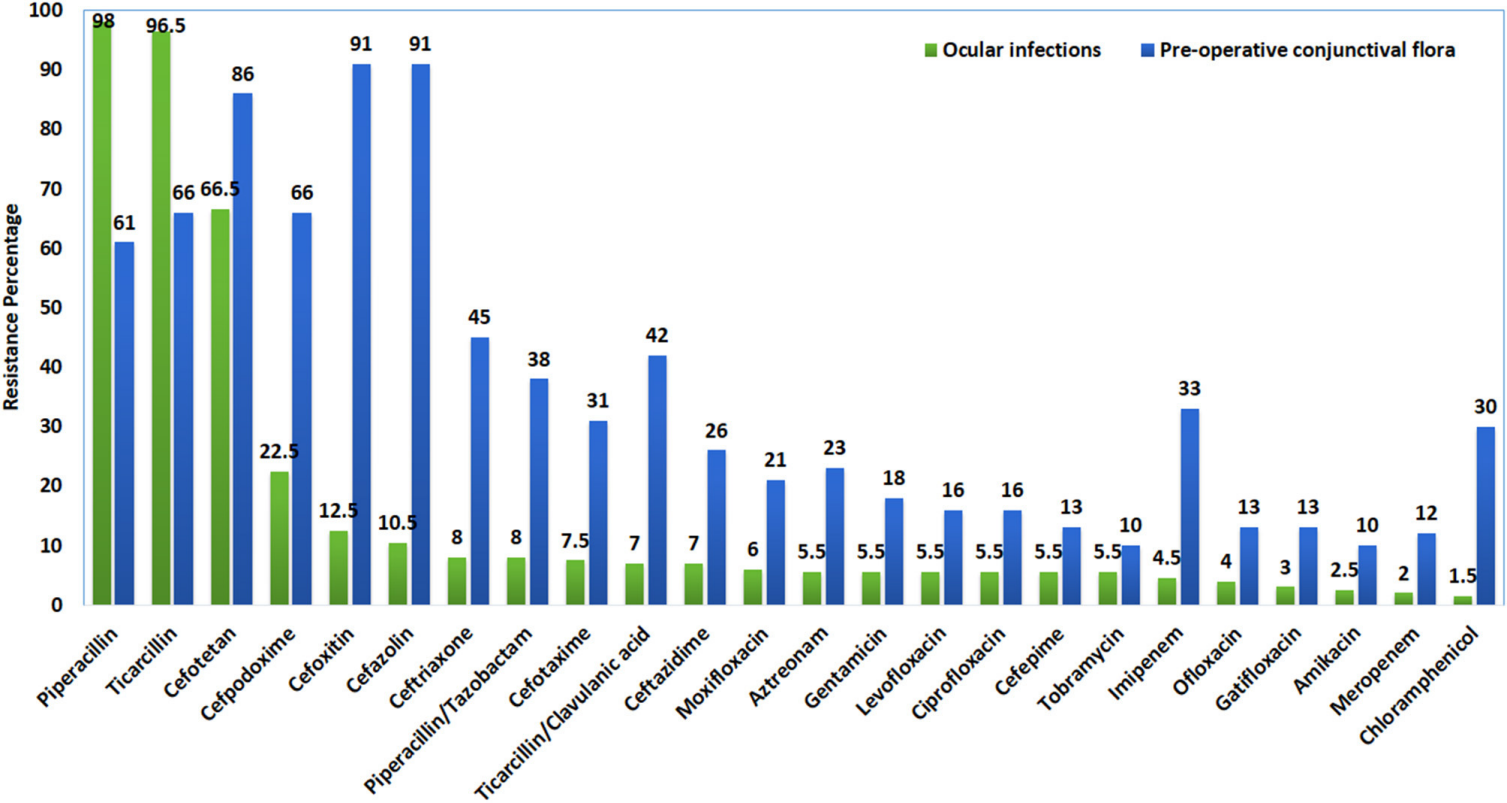
Comparative *in-vitro* antibiotic resistant profile of isolates from eye infections and pre-operative conjunctiva.

The antibiotic resistance profile of the 359 gram-negative bacterial isolates is presented in Table 3. *P. aeruginosa* isolates from both infection and control groups showed 100% resistance to cefoxitin, cefotetan, and cefpodoxime. In the carbapenem group, 29 and 23% were resistant to meropenem in both infection and control groups, respectively. In the fluoroquinolone group tested antibacterial which showed the resistance range from 30 to 34% in infection group and 14 to 18% in control group. *Enterobacteriaceae* isolates from infection showed 73% resistance to cefazolin, cefpodoxime, and piperacillin each at 67%. Whereas, in the control group, 72% of isolates were resistance to piperacillin and 62% to ticarcillin. In the carbapenem group, imipenem showed 30% resistance in both infection and control group *Enterobacteriaceae* isolates. The fluoroquinolone group tested antibacterial, which showed a resistance range of 18 to 33% in the infection group and 17 to 25% in the control group (Figure 1).

**Table 3:** *In-vitro* antibiotic susceptibility profile of Non-*Enterobacteriaceae* vs. *Enterobacteriaceae* from infection vs. control group.

**Name of the antibacterial agents**	**Non-** ***Enterobacteriaceae***								***Enterobacteriaceae***			
	**Infection group**				Control group*				**Infection group**		Control group*	
	***P*.**		**Other non-**		***P*.**		**Other non-**					
	***aeruginosa***		***Enterobacteriaceae***		***aeruginosa***		***Enterobacteriaceae***					
	**(*n =* 86)**	**%**	**(*n =* 41)**		**(*n =* 22)**	**%**	**(*n =* 37)**	**%**	***n =* 73**	**%**	***n =* 100**	**%**
**Carbapenems**												
Meropenem (10 mcg)	25	29	7	17	5	23	4	11	4	5	10	10
Imipenem (10 mcg)	9	10	10	24	0	0	22	59	22	30	30	30
**Fluroquinolones**												
Levofloxacin (5 mcg)	29	34	6	15	4	18	2	5	20	27	19	19
Moxifloxacin (5 mcg)	29	34	7	17	4	18	4	11	24	33	25	25
Ofloxacin (5 mcg)	28	33	5	12	4	18	0	0	13	18	17	17
Ciprofloxacin (5 mcg)	27	31	5	12	3	14	2	5	23	32	20	20
Gatifloxacin (5 mcg)	26	30	5	12	4	18	2	5	14	19	18	18
**Cephalosporins**												
**2nd generation**												
Cefoxitin (30 mcg)	86	100	39	95	22	100	35	95	30	41	22	22
Cefotetan (30 mcg)	86	100	35	85	22	100	18	49	12	16	10	10
**3rd generation**												
Cepfodoxime (10 mcg)	86	100	41	100	22	100	31	84	49	67	52	52
Ceftriaxone (30 mcg)	86	100	37	90	21	95	25	68	27	37	26	26
Cefotaxime (30 mcg)	57	66	21	51	10	45	13	35	31	42	26	26
Ceftazidime (30 mcg)	46	53	19	46	17	77	12	32	29	40	24	24

*Control group Preoperative conjunctival flora.

### Phenotypic Detection of MβLs Activity

**Figure 2:**
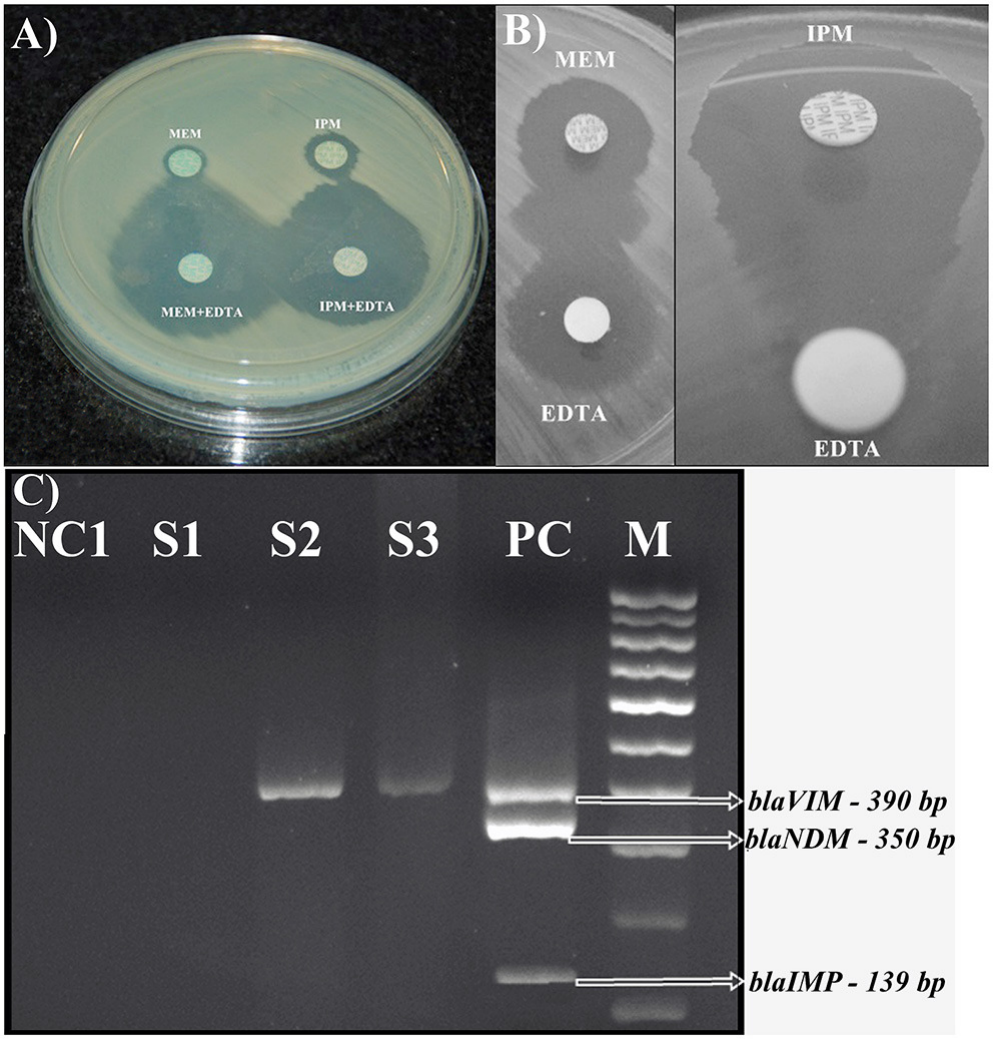
Phenotypic detection of MβL producing isolates **(A) CDT**, Combined disc test showing a positive result, the enhanced zone of inhibition around the imipenem + EDTA (10 μg+750 μg) (*Right below*) and meropenem + EDTA (10 μg+750 μg) (*Left below*) disc by ≥7 mm as compared to the imipenem (10 μg) (*Right*) and meropenem (10 μg) (*Left*) disc alone by a *bla*_VIM_ gene of *P. aeruginosa*. **(B) DDST**, Double disc synergy test portraying a synergistic inhibition zone between a meropenem disc (10 μg) (*Left side—Top*) and Imipenem disc (10 μg) (*Right side—Top*) and an EDTA disc (750 μg) (*Down both Right and left side*) as presented by a *bla*_VIM_ gene of *P. aeruginosa*. **(C)** Agarose gel electrophoresis (1.5%) used for the separation of multiplex PCR products. Results of multiplex PCR detecting *bla*_VIM_ genes; **Lane NC1 and NC2:** Blank controls. **Lane S1**—Negative amplification of MβLs genes. **Lane S2 and S3:** amplified products of *bla*_VIM_ genes among gram-negative bacterial isolates from preoperative conjunctival flora and ocular infections. **Lane PC**: amplified products of positive control strains carrying *bla*_VIM_ gene (390 bp), *bla*_IMP_ gene (162 bp), and *bla*_NDM_ gene (350 bp). **Lane MW**: Molecular weight marker 100-bp DNA ladder.

The production of MβLs was tested by a combined disc test and a double disc synergy test. Only 10.5% (21 of 200) and 0.6% (1 of 159) of the ocular infection and normal flora organisms exhibited MβL activity. Of which, 81% (17 of 21) were *P. aeruginosa*, 4.8% (1 of 21) were *Aeromonas hydrophila*, and *Acinetobacter lwoffii, Alcaligenes denitrificans*, and *Alcaligenes faecalis* each had 4.8% (1 of 21), respectively. Only one *A. hydrophila* isolate was positive for MβL producer from pre-operative conjunctiva. The highest number of MβLs producers was observed in isolates from vitreous fluid specimens (36%), followed by corneal scraping (18%), contact lens solution (14%) and conjunctival swab specimens (9%) (Supplementary Table 1 and Figures 2A,B).

Table 4 summarizes the results of the antibacterial susceptibility profile of MβL and non-MβL generating *P. aeruginosa* and Enterobacteriaceae. Out of the 108 (30%) *P. aeruginosa* isolates tested, 16% (17 of 108) were MβLs producers, and 84% (91 of 108) were non-MβL producers. Among the 17 MβL producing *P. aeruginosa* from infection group which showed the susceptibility to antibacterials were in the following order: aztreonam (59%), imipenem (47%), and cefepime (41%), while the resistance percentage to other antibacterials were in the following order: cefazolin (100%), cefpodoxime (100%), cefotetan (100%), ticarcillin (100%) and ticarcillin/clavulanic acid (100%), meropenem (100%), and gentamicin (94%). Of the 69 non-MβL producing *P. aeruginosa*, the susceptibility to antibacterials was in the following order: imipenem (100%), amikacin (90%), tobramycin (87%), and meropenem (88%). Hundred percentage of resistance was recorded for 2nd generation cephalosporins (cefoxitin and cefotetan), cefpodoxime, and ceftriaxone, and 99% of resistance was recorded for ticarcillin and ticarcillin with clavulanic acid. *P. aeruginosa* isolates (non-MβL producers) in the control group displayed the same resistant patterns as the infection group.

**Table 4:** Antibiotic resistant profile of MBL vs non-MBL producing *P.aeruginosa* and other *Enterobacteriaceae* vs non-*Enterobacteriaceae*.

**Name of the**	***Infection***				**Control**				**Infection group**				Control group*				**Non-MβL**			
**antibacterial**	***group***				group*				**Non-** ***Enterobacteriaceae***				**Non-** ***Enterobacteriaceae***				**producing**			
**agents**	***P. aeruginosa***				***P. aeruginosa***				**(Other than** ***P. aeruginosa*****)**				**(Other than** ***P. aeruginosa*****)**				***Enterobacteriaceae***			
	**MBL +ve**		**MBL –ve**		**MBL+ve**		**MBL –ve**		**MBL +ve**		**MBL –ve**		**MBL +ve**		**MBL –ve**		**Inf**		**Con**	
	***n =* 17**	**%**	***n =* 69**	**%**	***n =* 0**	**%**	***n =* 22**	**%**	***n =* 4**	**%**	**(*n =* 37)**	**%**	***n =* 1**	**%**	**(*n =* 36)**	**%**	**(*n =* 73)**	**%**	**(*n =* 100)**	**%**
**Carbapenems**	17	100	8	12																
Meropenem	17	100	8	12	0	0	5	23	4	100	3	8	1	100	3	8	4	5	10	10
(10 mcg)																				
Imipenem	9	53	0	0	0	0	0	0	4	100	6	16	1	100	21	58	22	30	30	30
(10 mcg)																				
**Fluroquinolones**	17	100	13	19			0													
Levofloxacin	17	100	13	19	0	0	4	18	1	25	5	14	0	0	2	6	20	27	19	19
(5 mcg)																				
Moxifloxacin	16	94	13	19	0	0	4	18	1	25	6	16	0	0	4	11	24	33	25	25
(5 mcg)																				
Ofloxacin	16	94	12	17	0	0	4	18	1	25	4	11	0	0	0	0	13	18	17	17
(5 mcg)																				
Ciprofloxacin	15	88	12	17	0	0	3	14	1	25	4	11	0	0	2	6	23	32	20	20
(5 mcg)																				
Gatifloxacin	15	88	11	16	0	0	4	18	1	25	4	11	0	0	2	6	14	19	18	18
(5 mcg)																				
**Cephalosporins**																				
**2nd generation**	17	100	69	100																
Cefoxitin	17	100	69	100	0	0	22	100	4	100	35	95	1	100	34	94	30	41	22	22
(30 mcg)																				
Cefotetan	17	100	69	100	0	0	22	100	2	50	33	89	1	100	17	47	12	16	10	10
(30 mcg)																				
**3rd generation**	17	100	69	100																
Cepfodoxime	17	100	69	100	0	0	22	100	4	100	37	100	1	100	30	83	49	67	52	52
(10 mcg)																				
Ceftriaxone	17	100	69	100	0	0	21	95	4	100	33	89	0	0	24	67	27	37	26	26
(30 mcg)																				
Cefotaxime	17	100	40	58	0	0	10	45	4	100	17	46	1	100	12	33	31	42	26	26
(30 mcg)																				
Ceftazidime	16	94	30	43	0	0	5	23	3	75	16	43	0	0	12	33	29	40	24	24
(30 mcg)																				
**4th generation**																				
Cefepime	10	59	10	14	0	0	2	9	3	75	10	27	0	0	2	6	16	22	14	14
(30 mcg)																				
**Monobactam**																				
Aztreonam	7	41	21	30	0	0	6	27	3	75	29	78	0	0	13	36	20	27	17	17
(30 mcg)																				
**Peniciliins and**	17	100	68	99																
**its combinations**																				
Ticarcillin	17	100	68	99	0	0	21	95	3	75	21	57	1	100	21	58	38	52	62	62
(75 mcg)																				

*Control group Preoperative conjunctival flora.

### Molecular Detection of MβL Genes

The Multiplex polymerase chain reaction assay detected 23% (83 of 359) of GNB isolates carrying only MβL encoding *bla*_VIM_ gene. There were 28% (56 of 200) *bla*_VIM_ gene-positive isolates from ocular infections and 17% (27 of 159) from pre-operative conjunctiva. The *bla*_VIM_ gene positive isolates were detected predominantly from 51.8% keratitis, 21.4% postoperative endophthalmitis, 17.9% conjunctivitis, and 34.1% from preoperative conjunctiva. Among the 56 isolates from ocular infections, 44 (78.6%) *bla*_VIM_ genes were detected in non-*Enterobacteriaceae* isolates and 12 (21.4%) among *Enterobacteriaceae* members. In the control group, 17 (63%) and 10 (37%), respectively, isolates were from the *Enterobacteriaceae* and non-*Enterobacteriaceae* families. A high prevalence of *bla*_VIM_ gene were detected from *P. aeruginosa* 42.9% (24 of 56) and *Alcaligens denitrificans* 10.7% (6 of 56) in the ocular infections and in the control group, a high number of *P. aeruginosa* and *E. coli*, each 14.8% (4 of 27), were detected (Supplementary Table 1 and Figure 2C).

**Figure 3:**
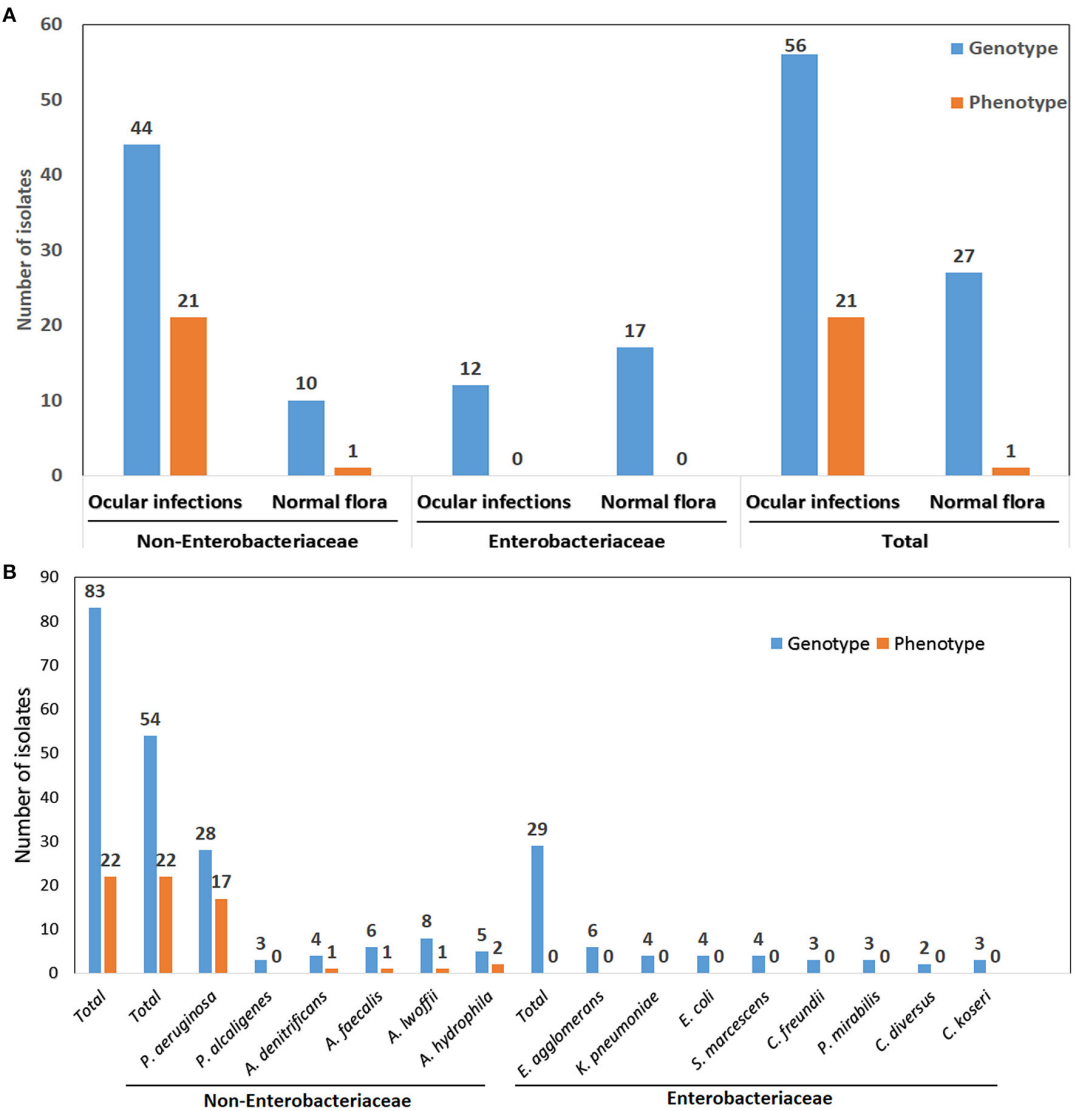
**(A)** Correlation of genotype and phenotype results of Non-*Enterobacteriaceae* and *Enterobacteriaceae* family isolates from various ocular infections and normal flora **(B)** Organisms wise correlation of genotype and phenotype results.

Despite the presence of genes in 83 organisms, only 22 isolates exhibited phenotypic expression. There were 54 genotypically positive isolates among non-*Enterobacteriaceae* members and 29 genotypically positive isolates among *Enterobacteriaceae* members. Even though they have the genes, none of the *Enterobacteriaceae* members express the phenotype in both the control and infection groups. Interestingly, among non-*Enterobacteriaceae* members, 21 phenotypically positive isolates belonged to the infection group, whereas only one isolate belonged to the control group (Figures 3A,B).

## Discussion

The ocular surface supports and maintains a small population of bacteria as normal flora with a combination of mechanical, anatomical, immunological, and microbiological factors (23). Previous studies reported the positive growth of microbes up to 83% in normal healthy conjunctiva (5), typically with coagulase-negative *Staphylococci* and occasionally with members of *Enterobacteriacae* (24). Another study showed 4.3% of gram-negative bacterial growth was seen among patients who underwent cataract or glaucoma surgery. (5). The current study also confirmed the colonization of gram-negative pathogens in the preoperative conjunctiva with *Enterobacteriaceae* family members accounted for 62.9, and 37.1% were non-*Enterobacteriaceae* families. *P. aeruginosa* (52.9%) was the most common among gram-negative bacteria in causing both internal and external eye infections (3). The current study also identified *P. aeruginosa* (43%) as the predominant pathogen causing eye infections.

In the present analysis, the *in-vitro* susceptibility profile of preoperative conjunctival GNB flora (control group) showed high level susceptibility to amikacin, tobramycin, meropenem, and ofloxacin, and high level resistance to cefoxitin, cefotetan, cefazolin, and ticarcillin. Studies from California have also shown a high level of resistance to cefazolin and a low level of resistance to amikacin, whereas none of the gram-negative bacterial isolates were resistant to the fluoroquinolone antibiotic group (24). In spite of the defense by the tears along with the flicker action of the eyelids, the resident bacteria can become opportunistic to cause infection, which necessitates the importance of prophylactic antibiotic interventions before ocular surgery.

Bacterial pathogens recovered from various ocular infections showed a high level of susceptibility to chloramphenicol and meropenem, followed by amikacin, gatifloxacin, and ofloxacin, while a high level of resistance was noted against piperacillin as reported in previous studies (25). The mechanism of resistance to β-lactams antibiotics can be mediated by multiple mechanisms, but the β-Lactamases production is a major resistance determinant in the gram-negative bacteria (26). The β-lactamase confers resistance to all β-lactam antibiotics and is susceptible to monobactam antibiotics, although inhibited by metal chelators like EDTA (27). The development of resistance can also occur via acquisition of plasmids or mobile genetic elements like integrons, which often co-exist with other resistance determinants (28). MβL producers have been found in a variety of health-care settings around the world, particularly in high-risk areas like intensive care units (ICUs), surgical wards, neonatal ICUs, and bone marrow transplantation units (28). Among the MβLs, the most prevalent types are class B and subgroup 3a beta-lactamases which include the plasmid-mediated genes of *bla*_IMP_ and *bla*_VIM_ found globally and frequently among non-fermenting bacteria, including *Enterobacteriaceae* (12).

There have been sporadic reports of MβLs from major hospitals in India, with some of them reporting prevalence rates as high as 75% (29). In systemic infection, *P. aeruginosa* (16–94.2%) and *Acinetobacter* spp. (14.2–70.9%) are the most common pathogens (19, 21, 29, 30). Similarly, the current study also identifies a high prevalence of MβLs producing *P. aeruginosa* (81%), and 4.8% of *Acinetobacter lwofffii* from eye infections. Previous finding on the MβLs producing ocular *Enterobacteriaceae* have shown 8%, which is higher when compared with the recent study from India presenting only 1.25% from ICUs and surgical wards (31). In this study, we have not detected a MβLs producer among ocular *Enterobactericeae* family isolates from both infection and control groups. MβL producing *P. aeruginosa* isolates showed susceptibility to ceftazidime (30%) and gentamicin (37.5%) (29), 56% of resistance and 13% of intermediate resistance to imipenem (32). In the present study, MβL producing *P. aeruginosa* isolates showed 53% resistance to imipenem and 100% resistance to meropenem.

The presence of the antibiotic resistant genes determines the prevalence of MβLs types, which varies greatly with geographical regions. In the present analysis, we found a high prevalence of 23% (83 of 359) *bla*_VIM_ type MβLs genes, with none representing *bla*_IMP_ and *bla*_NDM_ from preoperative conjunctival flora and various internal and external ocular infections. VIM-type enzymes were discovered in Verona, Italy, in 1997 (33), and 12 derivatives of the blaVIM-1 type of MβL gene have been reported worldwide to date (28). In terms of geographical distribution, the *bla*_VIM_ type gene has been found in *P. aeruginosa, K. pneumoniae, E. cloacae, P. mirabilis*, and *E. coli* isolates from Europe, South East Asia, and North America, and, more recently, India, Iran, and Australia (28).

The current study revealed 17% (27 of 159) isolates carried the *bla*_VIM_ gene among the preoperative conjunctival flora, while the remaining 28% (56 of 200) were the specimens obtained from the infected eye. In the current study, the *bla*_VIM_ gene was found in high abundance in *P. aeruginosa* and in low abundance in *Serratia marcescens, K. pneumoniae*, and *E. agglomerans*. Also, the current analysis highlights the presence of (*bla*_VIM_ type) MβLs mediated resistance genes among *P. aeruginosa* and *Enterobacteriaceae* family members. The high prevalence was detected predominantly in patients with keratitis and postoperative endophthalmitis, not with the normal flora. Compared to community and nosocomial-acquired infections, the prevalence currently determined is lower. (5).

There are multiple factors that determine the development of antibiotic resistance among the ocular isolates. The major concern is the use of higher antibiotic concentrations for treating ocular and preoperative prophylaxis than systemic antibiotics (34). The widespread use of broad spectral antibiotics, especially in non-infectious ocular cases, promotes the rate of selection pressures in antibiotic resistance (35), thus altering the characteristics of bacterial pathogens (36).

## Conclusion

To conclude, our findings fortified the fact that prevalence of the high resistance among the normal flora suggest the prophylactic measure is essential thus warranting a precisioned treatment in post-surgical period. Also, significant numbers of isolates having MβLs production also possess multidrug resistance. The emergence of MβLs-mediated resistance among ocular pathogens is a major problem for ophthalmologists since it limits the treatment options for treating ocular infections. Our study is only a preliminary report on the MβLs producing ocular gram-negative isolates from preoperative conjunctival flora and ocular infections, but it highlights the need for active surveillance. Large-scale multi-centric studies are required to understand the true nature of the prevailing antibiotic resistance pathogens. Monitoring the presence of drug resistance with detailed molecular prevalence will provide vital information for surveillance and help in evading these emerging mechanisms of antibiotic resistance. With the implication of advanced molecular methods like next generation sequencing, they will shed light on not only underlying molecular mechanisms but also aid in the development of better therapeutic targets.

## Data Availability Statement

The original contributions presented in the study are included in the article/Supplementary Material, further inquiries can be directed to the corresponding author/s.

## Ethics Statement

This study was approved by Aravind Eye Hospital Institutional Ethics Committee.

## Author Contributions

GR and VP researched data, wrote manuscript, and contributed to discussion. RD researched data and contributed to discussion. PL, ST, AA, and FS contributed to discussion and reviewed/edited manuscript. SR and MZ researched data, contributed to discussion, and reviewed/edited manuscript. TA reviewed/edited manuscript. PA and FA researched data and reviewed/edited manuscript. FH researched data, wrote manuscript, and reviewed/edited manuscript. All authors contributed to the article and approved the submitted version.

## Conflict of Interest

The authors declare that the research was conducted in the absence of any commercial or financial relationships that could be construed as a potential conflict of interest.

## Publisher's Note

All claims expressed in this article are solely those of the authors and do not necessarily represent those of their affiliated organizations, or those of the publisher, the editors and the reviewers. Any product that may be evaluated in this article, or claim that may be made by its manufacturer, is not guaranteed or endorsed by the publisher.
