# Supplementary material for: Prevalence and Molecular Characterization of Metallo β-Lactamase Producing Gram-Negative Pathogens Causing Eye Infections
Source: Front Public Health. 2022 Jun 14;10:870354. doi: 10.3389/fpubh.2022.870354 (PMC9237426; doi:10.3389/fpubh.2022.870354)
Supplement: Supplementary file 1 [file Table_1.pdf]

Supplementary Tables

Supplementary Table 1. Phenotypic and genotypic positive ocular Gram-negative pathogens from various eye infection and normal flora (control group)

Name of the bacterial isolates	**blaVIM-2 Genotype positive**				**MBL Phenotype Positive**			
	Infection group	%	Control group	%	Infection group	%	Control group	%
Non-Enterobacteriaceae	44	78.6	10	37	21	100	1	-
P. aeruginosa	24	42.9	4	14.8	17	81	-	-
P. alcaligenes	3	5.4	-	-	0	0	-	-
A. denitrificans	4	7.1	-	-	1	4.8	-	-
A. faecalis	6	10.7	-	-	1	4.8	-	-
A. lwoffii	5	8.9	3	11.1	1	4.8	-	-
A. hydrophila	2	3.6	3	11.1	1	4.8	1	100
Enterobacteriaceae	12	21.4	17	63	-	-	-	-
E. agglomerans	3	5.4	3	11.1	-	-	-	-
K. pneumoniae	3	5.4	1	3.7	-	-	-	-
E. coli	-		4	14.8	-	-	-	-
S. marcescens	4	7.1	-	-	--	-	-	-
C. freundii	2	3.6	1	3.7	-	-	-	-
P. mirabilis	-	-	3	11.1	-	-	-	-
C. diversus	-	-	2	7.4	-	-	-	-
C. koseri	-	-	3	11.1	-	-	-	-
**Total in numbers (%)**	56	100	27	100	21*	100	1	100

*n=21; 11 from postoperative endophthalmitis, 7 keratitis, and 3 contact-lens associated keratitis
